# Association between *NR3C1* gene polymorphisms and age-related hearing impairment in Qingdao Chinese elderly

**DOI:** 10.1186/s12920-021-01044-4

**Published:** 2021-07-28

**Authors:** Wanxue Song, Hainan Cao, Dongfeng Zhang, Haiyan Xu, Qianqian Zhang, Zhaoguo Wang, Suyun Li, Weijing Wang, Wenchao Hu, Bingling Wang, Haiping Duan

**Affiliations:** 1 Department of Epidemiology and Health Statistics, Public Health College, Qingdao University, No. 38 Dengzhou Road, Shibei District, Qingdao, 266021 Shandong Province People’s Republic of China; 2 Department of Otorhinolaryngology, Qingdao Municipal Hospital, Qingdao, 266011 Shandong Province People’s Republic of China; 3 Chengyang Street Community Health Service Center, No. 137 Wenyang Road, Chengyang District, Qingdao, 266109 Shandong Province People’s Republic of China; 4 Zaozhuang Municipal Center for Disease Control and Prevention, No. 223 Jiefang North Road, Shizhong District, Zaozhuang, 277100 Shandong Province People’s Republic of China; 5 Qingdao Municipal Center for Disease Control and Prevention, No. 175 Shandong Road, Shibei District, Qingdao, 266033 Shandong Province People’s Republic of China; 6 Qingdao Institute of Preventive Medicine, No. 175 Shandong Road, Shibei District, Qingdao, 266033 Shandong Province People’s Republic of China; 7 Department of Endocrinology, Qilu Hospital of Shandong University (Qingdao), No. 758 Hefei Road, Shibei District, Qingdao, 266033 Shandong Province People’s Republic of China

**Keywords:** Age-related hearing impairment, *NR3C1* gene, Weighted allele score

## Abstract

**Background:**

Age-related hearing impairment (ARHI) has attracted increasing attention recently. It is caused by genetic and environmental factors. A number of ARHI-related genes have been found. This study aimed to detect the potential association between *NR3C1* gene polymorphisms and ARHI by means of weighted allele score.

**Methods:**

A total of 861 participants from Qingdao city were selected by means of cluster random sampling. We statistically evaluated the characteristics of individuals and used the Mann–Whitney U test or chi-square test for comparison. The publicly available expression quantitative trait locus (eQTL) was queried on the website of the Genotype-Tissue Expression (GTEx). We used the weighted allele score and logistic regression analysis to explore the association between *NR3C1* gene polymorphisms and ARHI. Finally, the prediction model was constructed by logistic regression and receiver operating characteristic (ROC) curve.

**Results:**

All individuals over 60 years of age were enrolled in this study. The allele of rs61757411, rs41423247 and rs6877893 were significantly different between the ARHI group and the normal hearing group (*P* < 0.01). Though eQTL analysis, rs6877893 and rs33388 might affect the occurrence of ARHI by affecting the expression of *NR3C1* gene in artery aorta. Then we performed two models: one without adding any covariates into model and the other adjusting for demographic characteristic, smoking and drinking, diet and exercise, and physical conditions. In the multivariate-adjusted model 2, the odds ratio with 95% confidence interval for weighted allele score (*NR3C1*) was 0.841 (0.710–0.995, *P* = 0.043). The area under the ROC curve was 0.755, indicating that the model had good predictability.

**Conclusions:**

Our study suggests that *NR3C1* gene polymorphisms was significantly associated with ARHI.

## Introduction

Age-related hearing impairment (ARHI) is a kind of sensorineural hearing loss that shows a symmetrical bilateral and progressive hearing decline as people get older [1–3]. With the increase of human life expectancy and the proportion of elderly people, the prevalence of ARHI is increasing. In China, more than half of middle-aged and elderly people suffer from hearing impairment [3]. In a study in northern China, all participants over the age of 80 had hearing impairment [4]. More than 500 million people worldwide are estimated to suffer from ARHI by 2025 [5]. Personal daily communication and quality of life are affected by ARHI, leading to depression and social isolation [6, 7]. Hearing impairment has been demonstrated to be strongly linked to cognition decline that is considered an important factor for elderly people to live independently [8–10].

Medical science has allowed us to obtain knowledge on genetic predisposition and related disease factors in ARHI [11–15]. Genetic predisposition factors (such as gender, genes) and related disease factors (such as hypertension, diabetes, atherosclerosis, smoking, alcohol abuse, noise exposure, drugs) together contribute to the occurrence and development of ARHI [11, 13, 15–21]. Literature shows that 40%-50% of ARHI is related to genetic factors [22]. A study on twins and their family members showed that heritability was 0.7 or higher, which indicated the important role of genetic factors in hearing [18]. Genetic studies on humans have reported many ARHI-related genes, such as *GRM7*, *SIK3*, *ESRRG*, *SOD2*, *GSTM1*, *GRHL2*, *TRIOBP*, and a study have shown that *NR3C1* may be associated with sudden sensorineural hearing loss [2, 18, 23]. However, few studies have explored the relationship between *NR3C1* gene and ARHI.

Glucocorticoid receptor gene (nuclear receptor subfamily 3, group C, member 1, *NR3C1*) is located on human chromosome 5. This gene can encode glucocorticoid receptor (GR), which can combine with glucocorticoids (GC) to play various biological activities [24, 25]. Human and experimental animal studies have shown that GR is distributed in spiral ganglion neurons, spiral ganglion, stria vascularis, and organ of Corti, with the highest expression in the spiral ganglion of the inner ear [26–28]. When a genetic mutation occurs in *NR3C1* gene, it may affect the quantity and quality of GR. Moreover, GR in target tissues determines the biological response of these tissues to GC [29]. Therefore, GR is positively correlated with the degree of biological response of the inner ear to GC.

Variation of SNPs between different genes or multiple SNPs within the same gene may lead to the occurrence of ARHI [30]. However, the relationship between single SNP and ARHI is difficult to determine [31]. And the effect of single SNP is often very small, so the use of gene score can achieve higher detection efficiency. The allele score is a variable that integrates the variation information of SNPs in one gene and reflects the overall variation of the gene [32, 33]. Through allele score, we may find the relationship between whole variation information and disease at the gene level. Many studies had used the allele score to explore the relationship between genetic variation and disease, such as in diabetes, fasting glucose, subclinical atherosclerosis, and genetic variation that affects blood pressure and cardiovascular disease [34–36].

This study aims to explore whether *NR3C1* polymorphism is related to ARHI by using the weighted allele score. It is hoped that findings will provide a theoretical basis for revealing the mechanism of ARHI and the development of etiology, and provide a scientific basis for further expansion of research in the future.

## Research methods and procedures

### Study subjects

A cluster random sampling method was used to select two communities in Qingdao City, and the elderly in these two communities were used as participants. A total of 863 individuals participated in the initial survey according to the inclusion and exclusion criteria. Among them, two participants did not complete all questionnaires, corresponding to a participation rate of 99.77%. Finally, a total of 861 participants took part in the study. Because all our participants were elderly, we adopted face-to-face survey. The investigators were trained before investigation in order to improve the research quality of our questionnaire survey. The inclusion criteria were as follows: (1) age ≥ 60 years and older and (2) permanent Han Chinese residents in Qingdao (> 5 years). The exclusion criteria were as follows: (1) unable to cooperate and complete the listening test and questionnaire and (2) history of noise exposure, congenital hearing impairment, ear injury, head trauma, and use of ototoxic drugs.

### Hearing and Bone mineral density test

We first cleaned the external auditory canal, then looked at the eardrum and finally did a listening test. We arranged the participants to enter a quiet room (≤ 40 dB (A)) for a short rest. The air conduction hearing test was conducted by a professional audiometric doctor. A Pure Tone Audiometer (Orbiter 922, Madsen) and headset (TDH39) were utilized. Starting from the self-reported better hearing ear of the participants, pure tone air conduction thresholds of 0.5, 1.0, 2.0, and 4.0 kHz of the two ears were measured and the reading was accurate to 1 dB HL. According to the recommended criteria of the WHO (1997), the pure tone average (PTA) of the better hearing ear threshold at 0.5, 1.0, 2.0, and 4.0 kHz was used as the judgment standard of ARHI [1, 12]. So we divided the participants into normal hearing group (PTA ≤ 25 dB) and ARHI group (PTA > 25 dB) in accordance with this standard. The Bone mineral density test was performed by professionals using DXA Bone Densitometer commonly used.

### Genotyping and quality controls

We selected 9 SNP in the NR3C1 gene based on 1) refer to the results of our previous genome-wide association study (GWAS). 2) we searched on NCBI-SNP (https://www.ncbi.nlm.nih.gov/snp/), East Asian Samples of 1000 Genomes Project (https://www.ncbi.nlm.nih.gov/variation/tools/1000genomes/) and based on previous studies to selected target and functional SNPs of NR3C1 gene. SNPs must meet the minor allele frequency (MAF) greater than 0.05 in database of the Han Chinese in Beijing, China (CHB), and the minimum linkage disequilibrium correlation (r^2^) greater than 0.8. 3) through searching papers in the PubMed and Google, we carried out the literature research of validated hot SNPs. Based on the sequence information of SNPs, Assay Design 3.1 was used to design the PCR and single-base extension primer. Blood samples were collected and stored in a pre-prepared tube containing EDTA anticoagulant. White blood cells were isolated within 2 h after blood collection. DNA in venous blood samples was extracted by DNA extraction kit (BioTeke Corporation), and the OD value was detected with a NanoDrop 2000 spectrophotometer. After 1.25% agarose gel electrophoresis, the qualified DNA was transferred to a 96-well plate and stored at − 20 °C for future use. We obtained SpectroCHIP after PCR amplification, product alkaline phosphatase treatment, single-base elongation reaction, resin purification, and chip spot sample. SpectroCHIP was analyzed using matrix-assisted laser desorption/ionization time of flight (MALDI-TOF) mass spectrometer to obtain genotyping data. We referred to the East Asian Samples of 1000 Genomes Project and used the high-frequency allele as the major allele and the low-frequency allele as the minor allele.

### Calculation of weighted allele score

In order to improve the efficiency of *NR3C1*, weighted allele score was used to estimate the size of genetic effects. Weighted allele score is the number of SNP mutant allele in a gene multiplied by the corresponding weight and then add them together [37]. The weights were obtained from the current data using a tenfold cross-validation [38]. Specifically, we first divided the data into 10 samples randomly. However, the total number of our subjects was 861; thus, we randomly selected the number of the first nine samples as 86 and the 10th sample as 87. Then, we used the SNPs data of the first nine samples as the independent variable and the hearing levels as the dependent variable for logistic regression analysis. The obtained regression coefficients were used as the weights of the 10th sample. The weight calculation for the rest of the samples was the same.

The formula is as follows:

$$\mathrm{Allele score }= (\mathrm{W}1 \times \mathrm{ SNP}1+\mathrm{W}2 \times \mathrm{ SNP}2+\dots +\mathrm{Wi }\times \mathrm{ SNPi})/ (\mathrm{W}1+\mathrm{W}2+\dots +\mathrm{Wi})$$

i: the number of SNPs involved in constructing the allele score. SNP_i_: the number of minor alleles in a SNP. W_i_: the weight of each SNP.

### Covariates

The following covariates were included in this study: demographic characteristic (age, gender, osteoporosis awareness), smoking and drinking (smoking, drinking, passive smoking), diet and exercise (green tea, milk, frequency of fruit intake, spicy food, meal times, intake quantity, exercise duration), and physical condition (dizziness handicap inventory, antiosteoporosis medication, diabetes, hypertension, chronic liver disease, bone mineral density).

### Statistical analysis

We used the Kolmogorov–Smirnov normality test to verify the normality of continuous variables. Data that do not conform to the normal distribution were described in terms of median and quad ranges. First, we statistically evaluated the characteristics of individuals and used the Mann–Whitney U test or chi-square test for comparison. Each SNP was used as three categorical variables, and the wild type was used as the reference group. We determined the Hardy–Weinberg equilibrium (HWE) of each SNP. The publicly available expression quantitative trait locus (eQTL) was queried on the website of the Genotype-Tissue Expression (GTEx).

We used binary logistic regression analysis to explore the relationship between *NR3C1* gene polymorphisms and ARHI. We used two models: model 1without adjusting for any covariables and model 2 adjusting for demographic characteristic, smoking and drinking, diet and exercise, and physical conditions. We conducted logistic regression analysis with weighted allele score (*NR3C1*) as the independent variable and whether living with ARHI as the dependent variable. Finally, the prediction model was constructed by logistic regression and receiver operating characteristic (ROC) curve. Two-sided P-value less than 0.05 was considered statistically significant.

## Results

Compared with the normal hearing group, the ARHI group had significant differences in age, gender, drinking, smoking and PTA. The average age of participants in the ARHI group was higher than that in the normal hearing group. The prevalence of ARHI in males and females was 84.01% and 71.51%, respectively. The prevalence of ARHI was higher in males than in females. We also found that the prevalence of ARHI in non-drinkers and drinkers was 74.00%, 84.03%, respectively. The prevalence of ARHI in non-smokers and smokers was 72.73%, 84.08%, respectively (Table 1).

**Table 1:** Characteristics of participants by hearing levels

Variable	Hearing levels		*P*
	Normal hearing group	ARHI group	
PTA (dB HL)^1^	21.00 (5.00)	35.00 (15.00)	< 0.01
Age (years)^1^	65.00 (6.00)	68.00 (9.00)	< 0.01
Gender^2^			< 0.01
Male	55 (27.23)	290 (44.01)	
Female	147 (72.77)	369 (55.99)	
Drinking^2^			0.02
No	143 (70.79)	407 (61.76)	
Yes	59 (29.21)	252 (38.24)	
Smoking^2^			< 0.01
No	156 (77.23)	416 (63.13)	
Yes	46 (22.77)	243 (36.87)	
Diabetes^2^			0.26
No	33 (16.42)	132 (20.06)	
Yes	168 (83.58)	526 (79.94)	
Hypertension^2^			0.69
No	86 (42.57)	292 (44.31)	
Yes	116 (57.43)	367 (55.69)	
Chronic liver disease^2^			0.17
No	10 (4.95)	18 (2.73)	
Yes	192 (95.05)	641 (97.27)	
Bone mineral density^1^	− 2.30 (1.10)	− 2.40 (1.10)	0.63

^1^Data are presented as median (interquartile range) and Mann–Whitney U test

^2^Data are presented as N (%) and Person’s chi-square test

There were a total of eight SNPs and all SNPs are consistent HWE (Table 2).

**Table 2:** Hardy–Weinberg equilibrium (HWE) of each SNP

Gene	SNP	Allele	Genotype			*P*
			Wild type (N %)	Heterozygote (N %)	Homozygote (N %)	
*NR3C1*	rs6191	C > A	0 (0.00)	80 (9.30)	780 (90.70)	0.15
	rs61757411	G > T	494 (57.44)	321 (37.33)	45 (5.23)	0.44
	rs41400245	C > T	685 (79.56)	166 (19.28)	10 (1.16)	0.99
	rs258751	G > A	771 (89.76)	88 (10.24)	0 (0.00)	0.11
	rs6196	A > G	769 (89.52)	89 (10.36)	1 (0.12)	0.34
	rs41423247	G > C	551 (64.29)	271 (31.62)	35 (4.09)	0.82
	rs6877893	A > G	523 (60.88)	292 (33.99)	44 (5.13)	0.70
	rs12655166	T > C	658 (76.69)	189 (22.03)	11 (1.28)	0.53
	rs33388	T > A	525 (61.05)	288 (33.49)	47 (5.46)	0.37

There was no significant difference in the genotype of all SNP between the ARHI group and the normal hearing group (*P* > 0.05), as shown in Table 3.

**Table 3:** Genotype distribution of *NR3C1* compared between ARHI group and normal hearing group

SNP	ARHI group (N %)	Normal hearing group (N %)	*P*
rs6191			
AA	590 (89.76)	190 (94.06)	0.060
AC	68 (10.33)	12 (5.94)	
CC	0 (0.00)	0 (0.00)	
rs61757411			
GG	373 (56.69)	121 (59.90)	0.668
TG	251 (38.15)	70 (34.65)	
TT	34 (5.17)	11 (5.45)	
rs41400245			
CC	522 (79.21)	163 (80.69)	0.751
CT	130 (19.73)	36 (17.82)	
TT	7 (1.06)	3 (1.49)	
rs258751			
GG	590 (89.80)	181 (89.60)	0.935
AG	67 (10.20)	21 (10.40)	
AA	0 (0.00)	0 (0.00)	
rs6196			
AA	588 (89.50)	181 (89.60)	0.857
AG	68 (10.35)	21 (10.40)	
GG	1 (0.15)	0 (0.00)	
rs41423247			
GG	428 (65.24)	123 (61.19)	0.577
CG	202 (30.79)	69 (34.33)	
CC	26 (3.96)	9 (4.48)	
rs6877893			
AA	408 (62.10)	115 (56.93)	0.348
AG	218 (33.18)	74 (36.63)	
GG	31 (4.72)	13 (6.44)	
rs12655166			
TT	501 (76.26)	157 (78.11)	0.823
CT	147 (22.37)	42 (20.90)	
CC	9 (1.37)	2 (1.00)	
rs33388			
TT	411 (62.46)	114 (56.44)	0.298
AT	213 (32.37)	75 (37.13)	
AA	34 (5.17)	13 (6.44)	

The allele of rs61757411, rs41423247 and rs6877893 were significantly different between the ARHI group and the normal hearing group (*P* < 0.01), as shown in Table 4.

**Table 4:** The distribution of allele compared between ARHI group and normal hearing group

SNP	ARHI group (N %)	Normal hearing group (N %)	*P*
rs6191			
A	1248 (94.83)	392 (97.03)	0.067
C	68 (5.17)	12 (2.97)	
rs61757411			
G	997 (75.76)	312 (28.26)	< 0.001
T	319 (24.24)	792 (71.74)	
rs41400245			
C	1174 (89.07)	362 (89.60)	0.764
T	144 (10.93)	42 (10.40)	
rs258751			
G	1247 (94.90)	383 (94.80)	0.937
A	67 (5.10)	21 (5.20)	
rs6196			
A	1244 (94.67)	383 (94.80)	0.919
G	70 (5.33)	21 (5.20)	
rs41423247			
G	1074 (71.41)	315 (78.36)	0.005
C	430 (28.59)	87 (21.64)	
rs6877893			
A	1034 (67.49)	304 (75.25)	0.003
G	498 (32.51)	100 (24.75)	
rs12655166			
T	1149 (87.44)	356 (88.56)	0.552
C	165 (12.56)	46 (11.44)	
rs33388			
T	1035 (78.65)	303 (75.00)	0.123
A	281 (21.35)	101 (25.00)	

Our result showed that there was an association between rs33388 and ARHI in the additive model (OR = 0.731, 95%CI: 0.548–0.976, *P* = 0.034). Under the dominant model, individuals with (AT + AA) genotypes would have a reduced risk of having ARHI compared with individuals with TT genotypes (OR = 0.670, 95%CI: 0.468–0.961, *P* = 0.029). However, no other SNP were found.

Though eQTL analysis, we found that rs41423247, rs6877893, rs12655166 and rs33388 were the significant *cis*-eQTL in esophagus mucosa and artery aorta, as shown in Table 5. rs6877893 and rs33388 were associated with ARHI in artery aorta (*P* = 9.453 × 10^–5^ and *P* = 5.391 × 10^–5^).

**Table 5:** The result of expression quantitative trait locus (eQTL) study

SNP	Tissue	*P*	Gene
rs41423247	Esophagus Mucosa	9.453e−5	*NR3C1*
rs6877893	Artery Aorta	6.183e−5	*NR3C1*
rs12655166	Esophagus Mucosa	4.405e−10	*NR3C1*
rs33388	Artery Aorta	5.391e−5	*NR3C1*

The odds ratios (ORs) and 95% confidence intervals (95%CIs) between weighted gene score (*NR3C1*) and ARHI are shown in Table 6. In the logistic regression analysis of model 1 showed that there was no significant association of weighted allele score (*NR3C1*) with ARHI, and the OR (95%CI) was 0.971 (0.836–1.128, *P* > 0.05). However, the result changed that weighted allele score (*NR3C1*) was related with ARHI in model 2 and the OR (95%CI) was 0.841 (0.710–0.995, *P* = 0.043).

**Table 6:** Association of *NR3C1* gene score and ARHI

	Model 1			Model 2		
	OR	95% CI	*P*	OR	95% CI	*P*
Weight allele score (*NR3C1*)	0.971	0.836–1.128	0.700	0.841	0.710–0.995^*^	0.043*

*P < 0.05

**Fig. 1:**
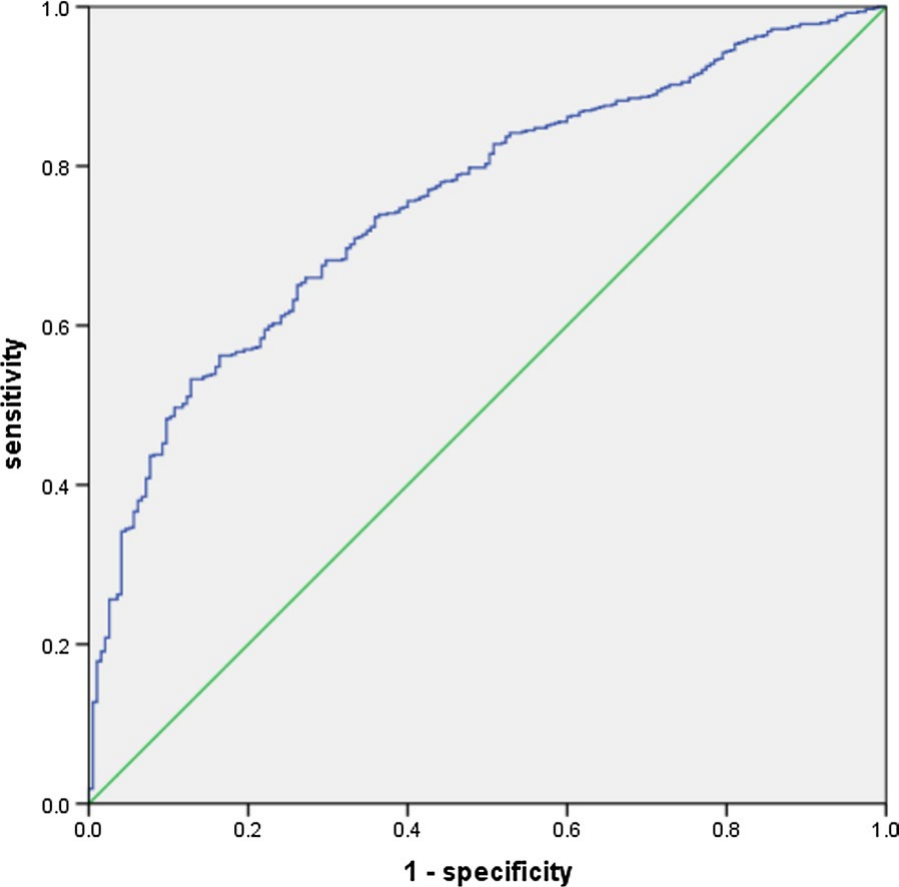
The receiver operating characteristic (ROC) curve of ARHI-related factors

Logistic regression was used to build the prediction model, and the area under the ROC curve was 0.755, indicating that the model had good predictability (Fig. 1).

## Discussion

GC have been widely used in the clinical treatment of various hearing disorders and achieved good efficacy since a long time ago [28]. The bioactivity of GC in body is produced by binding to the GC receptor (GR) encoded by *NR3C1*[39]. *BclI* polymorphism (*NR3C1,* rs41423247), a change in the downstream intron of exon 2, may affect an individual’s response to GC [40]. Furthermore, study have shown that *BclI* polymorphisms exhibit insulin resistance that is detrimental to hearing ability [41]. And our results suggested that the allele of rs41423247 was significantly different between the ARHI group and the normal hearing group. Though eQTL analysis, rs6877893 and rs33388 might affect the occurrence of ARHI by affecting the expression of *NR3C1* gene in artery aorta. Mice lacking GRs developed more severe atherosclerosis in the aorta [42]. And atherosclerosis has long been recognized as an important risk factor for ARHI [43].

The GR-mediated hearing protection may be related to the regulation of the expression of apoptosis-related proteins, which play an anti-apoptotic role [44]. GR encoded by *NR3C1* belongs to the nuclear hormone receptor superfamily and is distributed in the inner ear [26–28]. The protective effect of GC secreted by the hypothalamic–pituitary–adrenal axis (HPA) on hearing depends on GR [28]. Animal experiments showed that ARHI mice had apoptosis of hair cells in the inner ear and degenerative changes of auditory nerve [45, 46]. Inhibiting the expression of apoptosis-inducing factor (AIF) Fas or pro-apoptotic members in the Bcl-2 protein family and upregulating the expression of anti-apoptotic members of the Bcl-2 protein family exert an anti-apoptotic effect. For example, dexamethasone can resist the ototoxic effects of tumor necrosis factor-alpha (TNF-α), possibly by upregulating Bcl-2 and Bcl- × 1 through genomic action [47]. When the polymorphisms of *NR3C1* changes, it will not only affect the function of GR itself, but also affect the individual’s sensitivity to GC, thus leading to the occurrence of ARHI.

GC has significant anti-inflammatory and immunosuppressive effects and function through the GR signal pathway. Because the blood–labyrinth barrier (BLB) in the lateral wall of the cochlea strictly separates the cochlear microenvironment from the blood circulation, the inner ear has long been regarded as an organ that cannot be immunized. However, several studies have shown that there was an inflammatory reaction in the cochlea in participants with ARHI. Serum concentrations of TNF-α and C-reactive protein (CRP) are significantly increased in people with hearing impairment under the age of 60 [48]. Reactive oxygen species (ROS) was found in the cochlea of aged mice, and the levels of interleukin-1β, interleukin-6, interleukin-18 and TNF-α were significantly increased [49]. These may affect indicate that inflammation and immune response are important mechanisms for the occurrence and development of ARHI. After entering the nucleus, the GR-α, which plays a major anti-inflammatory role, binds to the GC response elements on the DNA, and its structure changes, affecting the transcription process and inhibiting the inflammatory response through the expression of anti-inflammatory proteins [50, 51]. Changes in *NR3C1* polymorphisms may affect the binding of GC and GR, which may result in the failure of the immune response and anti-inflammatory effects.

Our study has notable advantages. We used allele score, which integrates the overall situation of SNP variation inside a gene and reflects the overall variation information of the gene, so as to more comprehensively discuss the relationship between gene polymorphisms and disease at the gene level. However, this study also has a limitation. Because our study is cross-sectional, it is difficult to determine the cause and effect.

## Conclusions

This study of Qingdao Han Chinese elderly verifies the associations between *NR3C1* gene and ARHI.

## Data Availability

The datasets analysed during the current study are available in the online repositories. The names of the repository/repositories and accession number can be found below: https://pan.baidu.com/s/1gjE5bfeQO8zbccyLPaWDvw (extract code is 1234), 1gjE5bfeQO8zbccyLPaWDvw.

## Abbreviations

ARHI: Age-related hearing impairment
*NR3C1*: Nuclear receptor subfamily 3, group C, member 1
PTA: Pure tone average
GR: Glucocorticoid receptor
MAF: Minor allele frequency
HWE: The Hardy–Weinberg equilibrium
HPA: The hypothalamic–pituitary–adrenal axis
